# Pseudogene fms-related tyrosine kinase 1 pseudogene 1 (FLT1P1) cooperates with RNA binding protein dyskeratosis congenita 1 (DKC1) to restrain trophoblast cell proliferation and angiogenesis by targeting fms-related tyrosine kinase 1 (FLT1) in preeclampsia

**DOI:** 10.1080/21655979.2021.1988366

**Published:** 2021-10-26

**Authors:** Zhenjing Chi, Yanlan Sun, Zhou Yu, Fenmei Zhou, Hairong Wang, Muling Zhang

**Affiliations:** Department of Obstetrics, The Affiliated Huaian No. 1 People’s Hospital of Nanjing Medical University, Huaian, Jiangsu, China

**Keywords:** *FLT1P1*, *DKC1*, *FLT1*, preeclampsia

## Abstract

In preeclampsia (PE), preexistent maternal endothelial dysfunction leads to impaired placentation and vascular maladaptation. Long noncoding RNAs (lncRNAs) have been shown to participate in the placentation process. LncRNA fms-related tyrosine kinase 1 pseudogene 1 (*FLT1P1*) was previously reported to be upregulated in PE. In this study, we verified the effect of *FLT1P1* and its cognate gene *FLT1* on trophoblast cell proliferation and angiogenesis by using Cell Counting Kit-8 (CCK-8) assay, tube formation assay, and western blot analysis. Their binding to RNA binding protein dyskeratosis congenita 1 (*DKC1*) was validated by conducting RNA immunoprecipitation (RIP) and RNA pulldown assays. In this study, knockdown of *FLT1P1* or *FLT1* was found to promote cell proliferation and angiogenesis in trophoblasts. In addition, *FLT1P1* recruited *DKC1* to stabilize *FLT1*. Importantly, silencing *FLT1P1* or *DKC1* decreased the stability of *FLT1*. Rescue assays revealed that *FLT1* overexpression reversed the effect of silenced *FLT1P1*. Overall, *FLT1P1* cooperates with *DKC1* to restrain trophoblast cell proliferation and angiogenesis by targeting *FLT1*.

## Introduction

Preeclampsia (PE), with a global prevalence of estimated 2% to 8% of all pregnancies, is one of the causes of maternal and fetal mortality [1]. It is related to abnormal placentation and maladaptation of the maternal cardiovascular system, in which vascular resistance is abnormally increased, resulting in maternal hypertension [2]. Importantly, the disease is linked to maternal long-term cardiovascular disorders and systemic inflammation [3]. PE is a consequence of various pathophysiological processes including altered trophoblast proliferation, reduced invasion of extravillous trophoblasts, impaired differentiation of trophoblastic cells, and dysregulated immunoregulation [4,5]. However, its pathogenesis and associated molecular mechanisms are still not completely understood.

Long non-coding RNAs (lncRNAs) are defined as transcribed RNA molecules ranging from 200 to 100,000 nucleotides that do not code for any protein [6]. It has been recognized that lncRNAs play different roles in many important biological processes, including regulation of transcriptional and posttranscriptional processes, epigenetic control, differentiation and development, cell cycle control, apoptosis, and metabolic processes [7], participating in the pathogenesis and development of various diseases, including PE [8,9]. Recently, lncRNAs were identified to be associated with the pathogenesis of PE. For example, downregulation of lncRNA maternally expressed gene 3 (*MEG3*) promotes the apoptosis and suppresses the migration of trophoblast cells [10]. LncRNA metastasis associated lung adenocarcinoma transcript-1 (*MALAT-1*) is expressed at a low level in PE and regulates JEG-3 trophoblast cell migration and invasion [11]. As a subtype of lncRNA, pseudogene shares high sequence homology with its cognate gene, which has the capacity to code protein [12]. It was also found that pseudogene could regulate its cognate gene. As reported, the pseudogene phosphatase and tensin homolog pseudogene 1 (*PTENP1*) modulate the level of its matched protein-coding gene phosphatase and tensin homolog (*PTEN*) [13]. Since then, numerous pseudogenes have been validated to exert critical functions in diverse pathophysiological and physiological processes [14,15]. There are few reports concerning the roles of pseudogenes in PE. The pseudogene urate (hydroxyiso-) hydrolase, pseudogene (*URAHP*), promotes proliferation and regulates the pathogenesis of PE [16]. Dysfunction of pseudogene phosphoglycerate kinase 1, pseudogene 2 (*PGK1P2*) is involved in PE by acting as a competing endogenous RNA of phosphoglycerate kinase 1 [17]. The structural similarity to lncRNA makes the pseudogene modulate its target gene expression by recruiting RNA binding proteins (RBPs) at the transcriptional level [18,19]. Dyskeratosis congenita 1 (*DKC1*) is a nucleolar, RBP, which is highly conserved in eukaryotes [20]. This protein is a key component of the telomerase complex and an essential structural subunit of the telomerase ribonucleoprotein and it has the ability to activate telomerase ribonucleoprotein activity, resulting in telomere shortening [21]. Previous studies have shown that *DKC1* as a RBP is negatively regulated by *MEG3* that has been frequently reported to promote trophoblast migration and decrease apoptosis in PE [22–24]. We found a novel lncRNA, *FLT1P1* (fms-related tyrosine kinase 1 pseudogene 1), as the pseudogene of *VEGFR1* (vascular endothelial growth factor receptor-1), which is also known as *FLT1* (fms-related tyrosine kinase 1) [25]. In addition, *FLT1P1* was reported to be overexpressed in preeclamptic placentas [26], but its function and molecular mechanism remain unclear in PE.

In this study, we focused on identifying the role of the pseudogene *FLT1P1* in trophoblast cell proliferation and angiogenesis and mechanistically analyzing how *FLT1P1* exerts its function in PE. We hypothesized that *FLT1P1* may exert its function in PE by interacting with the RBP *DKC1* to stabilize its cognate gene *FLT1*. Our study may provide a novel regulatory mechanism for exploration of the pathogenesis of PE.

## Materials and methods

### Samples

Normal placentas (n = 10) were obtained from full-term births after the cesarean section. Age-matched placentas were obtained from women with severe PE (n = 10) after cesarean section. All placentas involved in this study were collected by procedures of planned cesarean section without the aid of artificial labor. Placental tissues were obtained by a certified doctor by making a vertical incision across a normal area at the center, involving fetal and maternal placental surfaces. Tissues having calcified deposits or clots were excluded. The experiments were approved by the Ethics Committee of the Affiliated Huaian No. 1 People’s Hospital of Nanjing Medical University. All volunteers participating in this study signed written informed consent.

### Isolation and culture of human primary trophoblast cells (PTBs)

Term placentas were collected from uncomplicated pregnancies after cesarean delivery. Isolation and culture of PTBs was done according to conventional methods. Specifically, we used a protocol based on the classic trypsin digestion and Percoll gradient centrifugation method, as previously described [27]. Briefly, the placental tissues were washed, sheared, weighed, and digested in a solution containing 0.125% trypsin and 0.03% DNase (Sigma, USA). The supernatant was collected, and the pellet was kept. This process was performed in order to discard the outer syncytium and to keep the underlying trophoblasts. The pellet was then purified with 5%–65% Percoll density gradients (Sigma), which allowed the collection of the trophoblasts. Finally, the trophoblasts were cultured in Dulbecco’s modified Eagle’s medium (Thermo Fisher Scientific, USA) containing 10% fetal bovine serum (FBS).

### Cell culture

HTR-8/SVneo (HTR8) and BeWo cell lines were obtained from the Type Culture Collection of the Chinese Academy of Sciences (Shanghai, China). Cells were cultured in Roswell Park Memorial Institute (RPMI) 1640 (HyClone, USA) supplemented with 100 U/ml penicillin (HyClone), 10% heat-inactivated FBS, and 100 μg/ml streptomycin (Invitrogen, USA) in a humidified incubator at 37°C with 5% CO_2_.

## Cell transfection

The short hairpin RNAs (shRNAs) against *FLT1P1, FLT1* or *DKC1* (sh-*FLT1P1*, sh-*FLT1* or sh-*DKC1*) and the scrambled negative control (sh-NC) were designed and synthesized by GenePharma (Shanghai, China). The plasmid vector expressing full-length *FLT1* was generated by GenePharma to overexpress *FLT1* and termed as pcDNA3.1/*FLT1* (*FLT1*), and the empty vector (vector) was used as a negative control. Cells were seeded in 24-well plates at 2 × 10^5^ cells/well and transfected with 40 nM shRNA vector or 0.2 μg overexpression vector following the instructions of Lipofectamine 2000 (Invitrogen, USA) as described previously [28], and cells were harvested at 48 h for further analysis.

## Reverse transcription-quantitative polymerase chain reaction (RT-qPCR)

TRIzol (Invitrogen, USA) was used to isolate total RNA from trophoblast cells. Then, the first-strand cDNA was generated by ImProm-II Reverse Transcription System (Promega, USA). RT-qPCR was conducted by using SYBR Green qPCR assay (Takara, Dalian, China) and gene-specific primers. The relative gene expression was calculated by the 2^−ΔΔCt^ method [29], and glyceraldehyde-3-phosphate dehydrogenase (GAPDH) was used as the internal control. *FLT1P1*, forward: 5ʹ-AAGAACGCCGATTATGTGAG-3ʹ, reverse: 5ʹ-CAAGAGCCACCCATTTCAG-3ʹ; *FLT1*, forward: 5ʹ-CAAGATTGACTTGAGAGTAACCAG-3ʹ, reverse: 5ʹCTGGAATGGCAGAAACTGG-3ʹ; *DKC1*, forward: 5ʹ-GGTATAGTAGCCAAGATCAAGAG-3ʹ, reverse: 5ʹ-TTCTGACTTGCCTTTGGAC-3ʹ; GAPDH, forward: 5ʹ-TCAAGATCATCAGCAATGCC-3ʹ, reverse: 5ʹ-CGATACCAAAGTTGTCAT GGA-3ʹ.

## Cell counting kit-8 (CCK-8) assay

The cell Counting Kit-8 (CCK-8, Dojindo Molecular Technologies, Kyushu, Japan) was used to measure the viability of stably transfected trophoblast cells. In brief, cells were seeded in 96-well plates at 4 × 10^3^ cell/well. At 24 h, 48 h, and 72 h, each well was added with 10 μl CCK-8 solution for an additional 1 h of incubation at 37°C. Finally, a microplate reader (BioRad, CA, USA) was used to read the absorbance value at 450 nm [30].

### Tube formation assay

For the tube formation assay, as described previously [31], 24-well plate was coated with growth factor reduced Matrigel (60 µl, Corning, NY, USA) for 1 h at 37°C. A total of 1 × 10^5^ trophoblast cells in a medium containing 10% FBS were plated on top of presolidified Matrigel. Once seeded on Matrigel, capillary tubes and networks start to form. After 6 h of incubation, plates were examined with a microscope (Nikon, Japan), and images were taken. The number of branching points was quantified using ImageJ plug-in according to the protocol angiogenesis analyzer from Gilles Carpentier.

## Western blot analysis

Western blot was performed as previously published [32]. Total protein was extracted from cells using radioimmunoprecipitation assay lysis buffer (Life Technologies, USA) containing protease inhibitors (Sigma). After that, protein concentration was quantified with the Bicinchoninic Acid Assay (Beyotime, China). Before transferring into polyvinylidene difluoride membranes (Millipore, Billerica, MA, USA), the protein samples were separated with 10% sodium dodecyl sulfate-polyacrylamide gel electrophoresis. After blocking with 5% skim milk, the membranes were incubated with primary antibodies including vascular endothelial growth factor A (VEGFA; ab214424, 1:1000, Abcam, Cambridge, USA), fibroblast growth factor 2 (FGF2; ab208687, 1:1000), transforming growth factor beta (TGF-β; ab124894, 1:1000), *FLT1* (ab32152, 1:1000), and GAPDH (ab181602, 1:10,000) overnight at 4°C. After washing, the membranes were further incubated with secondary antibodies (ab205718, 1:3000) for 1 h at room temperature. The blots were detected by using a chemiluminescence substrate (Pierce, USA). Immunoblot signals were quantified using Image Quant software (GE Healthcare).

### RNA immunoprecipitation (RIP) assay

For the RIP assay, the Magna RIP TM RNA-Binding Protein Immunoprecipitation Kit (Millipore, Billerica, USA) was used following the manufacturer’s protocols [28]. The trophoblast cells at 80–90% confluency were scraped off and then lysed in a complete radioimmunoprecipitation assay lysis buffer. A total of 100 μl of the cell extract were incubated with anti-*DKC1* or control IgG (Millipore)-conjugated magnetic beads at 4°C for 6 h, and anti-IgG was used as a negative control. The beads were then washed with a washing buffer and the complexes were incubated with Proteinase K for 30 min at 55°C to remove the protein. Finally, immunoprecipitated RNA was purified and analyzed by RT-qPCR.

## RNA pulldown assay

As described previously [33], biotin-labeled *FLT1P1* and its antisense RNA were transcribed in vitro based on the corresponding PCR product using T7 RNA polymerase (Ambio Life) and Biotin RNA Labeling Mix (Roche, Mannheim, Germany). The obtained product was purified using RNeasy Plus Mini Kit (Qiagen). For the pulldown assay, 20 µl Dynabeads M-280 Streptavidin beads (Thermo Fisher Scientific, MA, USA) were activated and blocked with 10 µg/ml RNase-free BSA and yeast tRNA (Sigma) for 30 min at 4°C. Cell lysates were incubated with the beads at room temperature for 2 h, followed by pulldown, RNA extraction, and subsequent RT-qPCR quantification.

### RNA stability assay

For detecting *FLT1* mRNA stability, trophoblast cells were treated with 10 µg/ml actinomycin D (Sigma) after transfecting with sh-*FLT1P1* or sh-*DKC1* [34]. At 3 h, 6 h, and 9 h, cells were collected, and RNA extraction was performed by using TRIzol reagent (Invitrogen). RT-qPCR was used to measure the *FLT1* mRNA level.

### Statistical analysis

Statistical analysis was performed using Graphpad Prism 5 software (GraphPad, USA), and the results are presented as the mean ± standard deviation (SD). Each assay was repeated in triplicate. A comparison between groups was performed using Student’s *t* test or one-way or two-way analysis of variance (ANOVA). P value less than 0.05 was considered statistically significant.

## Results

In this study, we focused on identifying the role of the pseudogene *FLT1P1* in trophoblast cell proliferation and angiogenesis and mechanistically analyzing how *FLT1P1* exerts its function in PE. We hypothesized that *FLT1P1* may exert its function in PE by interacting with the RBP *DKC1* to stabilize its cognate gene *FLT1*. Our results showed that *FLT1P1* and *FLT1* play a vital role in PE by regulating trophoblast cell proliferation and angiogenesis. Moreover, *FLT1P1* increases *FLT1* mRNA stability via recruiting *DKC1*.

### FLT1P1 *knockdown promotes the proliferation and angiogenesis in trophoblast cells*

**Figure 1.**
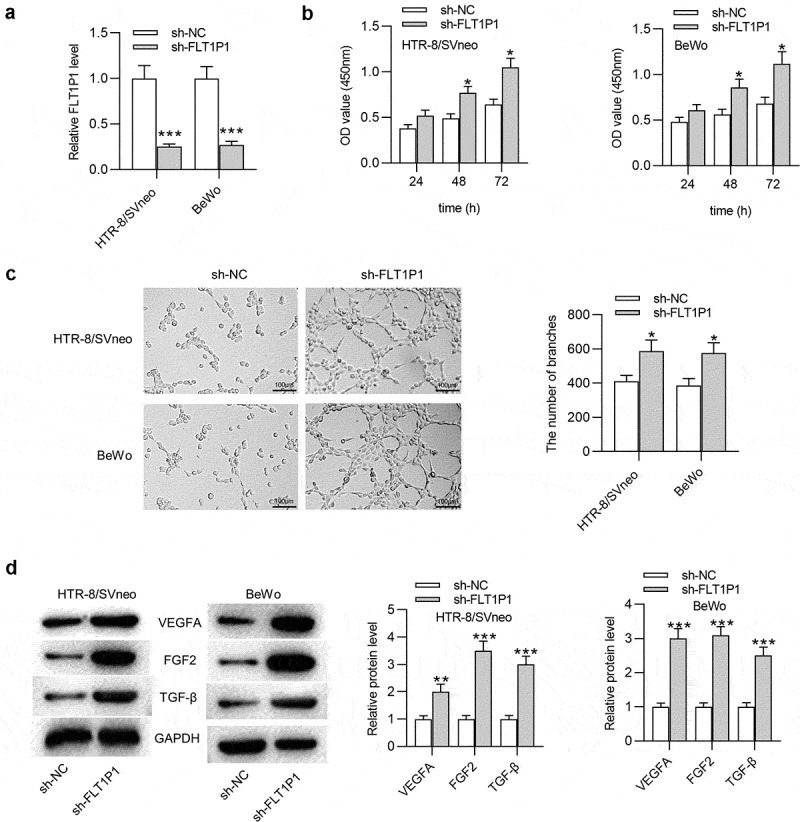
***FLT1P1* knockdown promotes trophoblast cell proliferation and angiogenesis**. (a) *FLT1P1* expression in trophoblast cells transfected with sh-*FLT1P1* or sh-NC was measured by RT-qPCR. (b) The proliferation of HTR8 and BeWo cells after *FLT1P1* knockdown was detected by CCK-8 assay. (c) The angiogenesis in sh-*FLT1P1*-transfected HTR8 and BeWo cells was assessed by tube formation assay. (d) Western blot analysis was performed to measure the VEGFA, FGF2 and TGF-β protein levels in HTR8 and BeWo cells transfected with sh-*FLT1P1* or sh-NC. *p < 0.05, ***p < 0.001

Human PTBs were isolated from PE and healthy placentas. The results of RT-qPCR showed that FLT1P1 was upregulated in PTBs isolated from PE patients compared with healthy controls (Fig. S1A). Next, we investigated the biological relevance of *FLT1P1* in PE progression using two trophoblast cell line (HTR8 and BeWo). As shown in Figure 1a, the expression of *FLT1P1* in HTR8 and BeWo cells was effectively knocked down by transfection with the specific shRNA (sh-*FLT1P1*) compared to the sh-NC. Next, the functional role of *FLT1P1* in trophoblast cell proliferation and angiogenesis was assessed by CCK-8 assay, tube formation assay, and western blot analysis. According to CCK-8 assay, the proliferative ability of trophoblast cells was increased after knocking down *FLT1P1* (Figure 1b). The results of the tube formation assay showed that *FLT1P1* downregulation significantly increased the number of junctions and nodes in HTR8 and BeWo cells (Figure 1c). Furthermore, the expression levels of angiogenesis-associated markers (VEGFA, FGF2 and TGF-β) was evaluated using western blot. As shown in Figure 1d, the expression levels of VEGFA, FGF2, and TGF-β were all increased in the presence of sh-*FLT1P1* (Figure 1d). Overall, these findings showed that *FLT1P1* knockdown increases the proliferation and angiogenesis in trophoblast cells.

### FLT1 *knockdown promotes trophoblast cell proliferation and angiogenesis*

**Figure 2.**
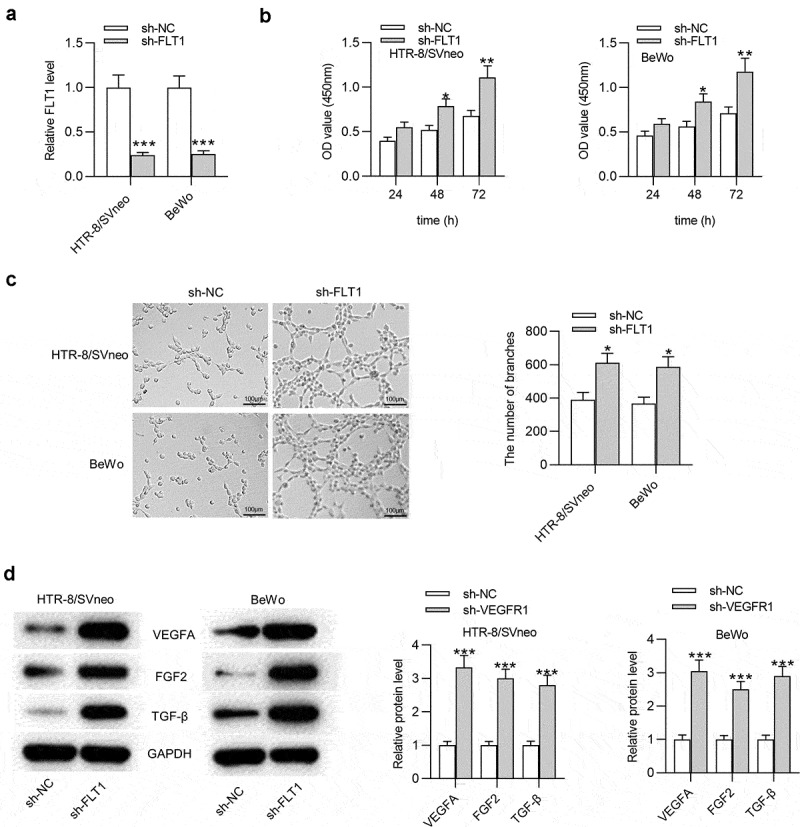
***FLT1* knockdown promotes the proliferation and angiogenesis in trophoblast cells**. (a) *FLT1* expression in trophoblast cells transfected with sh-*FLT1* or sh-NC was measured by RT-qPCR. (b) CCK-8 assay was performed to assess the proliferation in sh-*FLT1*-transfected HTR8 and BeWo cells. (c) The angiogenesis in HTR8 and BeWo cells after *FLT1* knockdown was evaluated by tube formation assay. (d) The VEGFA, FGF2 and TGF-β protein levels in sh-*FLT1*-transfected HTR8 and BeWo cells were measured by western blot analysis. *p < 0.05, **p < 0.01, ***p < 0.001

As the cognate gene of *FLT1P1*, the biological role of *FLT1* in trophoblast cells was investigated. The results of RT-qPCR and western blot showed that the FLT1 mRNA and protein levels were upregulated in PTBs isolated from PE patients compared with healthy controls (Fig. S1B-1 C). The sh-*FLT1* was transfected into HTR8 and BeWo cells to silence *FLT1*. The results of RT-qPCR showed that *FLT1* expression was significantly downregulated in the sh-*FLT1*-transfetced HTR8 and BeWo cells (Figure 2a). CCK-8 assay manifested that sh-*FLT1* transfection caused an increase in cell proliferation (Figure 2b). Next, the angiogenesis ability after sh-*FLT1* transfection was evaluated by tube formation assay. The results demonstrated that the angiogenesis in HTR8 and BeWo cells was promoted after silencing *FLT1* (Figure 2c). Additionally, the results of the western blot indicated that *FLT1* knockdown contributed to elevated protein levels of VEGFA, FGF2 and TGF-β in HTR8 and BeWo cells (Figure 2d). In conclusion, *FLT1* knockdown increases trophoblast cell proliferation and angiogenesis.

### DKC1 *acts as an RBP for* FLT1P1 *and* FLT1

**Figure 3.**
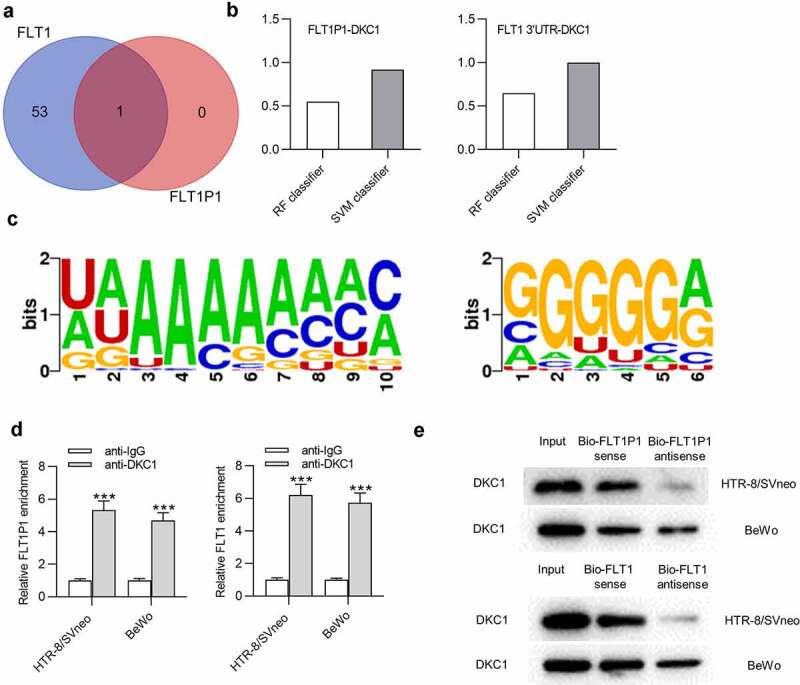
**The interaction between *DKC1* and *FLT1P1* (or *FLT1*)**. (a) The predicted RBP for *FLT1P1* and *FLT1* was obtained in starBase. (b) The prediction of the interaction probabilities of *FLT1P1* or *FLT1* with *DKC1* by RPISeq (http://pridb.gdcb.iastate.edu/RPISeq/). Predictions with probabilities > 0.5 are considered ‘positive’, indicating that the corresponding RNA and protein are likely to interact. (c) The motif of *DKC1* in *FLT1P1* and *FLT1*. (d-e) The binding of *DKC1* to *FLT1P1* (or *FLT1*) was validated by RIP and RNA pull down assays. ***p < 0.001

Next, we investigated whether there is a regulatory mechanism between *FLT1P1* and *FLT1*. Evidence has confirmed that the pseudogene could recruit RBPs to modulate its target gene expression [18,19]. Thus, we sought to identify the common RBP for *FLT1P1* and *FLT1*. At the starBase website (http://starbase.sysu.edu.cn/), *DKC1* was predicted as an RBP that has the potential to interact with *FLT1P1* and *FLT1* (Figure 3a). Then, the binding capacity between *FLT1P1* (or *FLT1*) and *DKC1* was further predicted at the RPISeq website (http://pridb.gdcb.iastate.edu/). The score of RF Classifier is over 0.5 and the score of SVM Classifier is over 0.8, indicating that *FLT1P1* (or *FLT1*) stands a good chance of binding to *DKC1* (Figure 3b). As predicted in starBase, the motif of *DKC1* in *FLT1P1* and *FLT1* was obtained (Figure 3c). RIP assay showed that *FLT1P1* and *FLT1* were both enriched in the beads conjugated with anti-*DKC1* compared to anti-IgG in trophoblast cells (Figure 3d). To further investigate the interaction between *FLT1P1* (or *FLT1*) and *DKC1*, a biotin-labeled *FLT1P1* (or *FLT1*) probe was used to perform RNA pulldown assay in HTR8 and BeWo cells. The results revealed that the *DKC1* was significantly enriched in the *FLT1P1* (or *FLT1*) sense probe compared with the sense probe (Figure 3e). These results suggested that *FLT1P1* (or *FLT1*) could bind to *DKC1*.

## *FLT1P1* increases *FLT1* mRNA stability by recruiting *DKC1*

**Figure 4.**
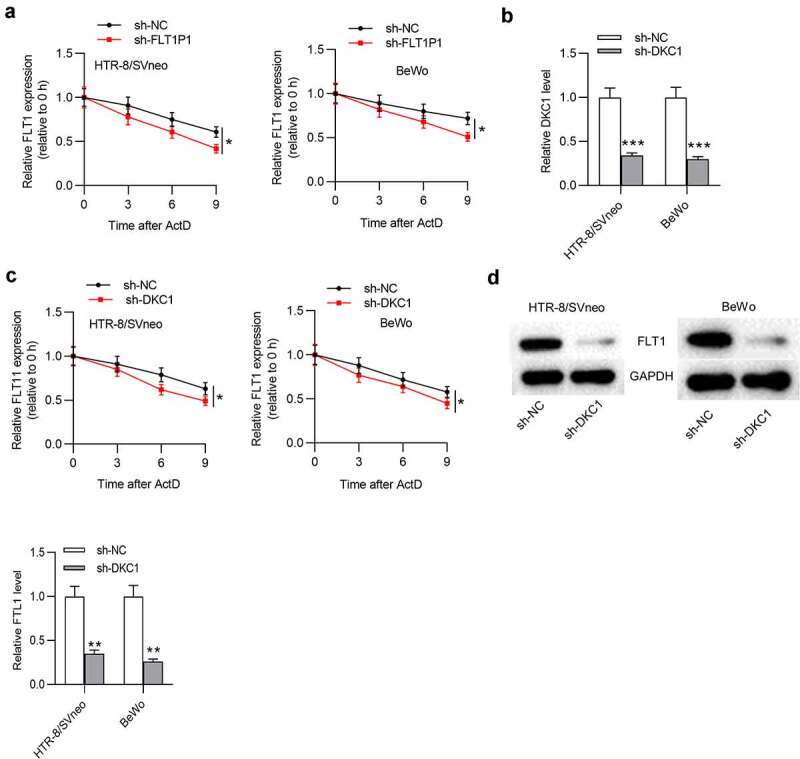
***FLT1P1* increases *FLT1* mRNA stability by recruiting *DKC1***. (a) *FLT1* mRNA expression under actinomycin D treatment in the sh-*FLT1P1* and sh-NC groups was measured by RT-qPCR. (b) The transfection efficiency of sh-*DKC1* was assessed by RT-qPCR. (c) *FLT1* mRNA expression under actinomycin D treatment in the sh-*DKC1* and sh-NC groups was measured by RT-qPCR. (d) The *FLT1* protein level in trophoblast cells transfected with sh-*DKC1* was measured by western blot analysis. *p < 0.05, **p < 0.01, ***p < 0.001

Subsequently, to test whether *FLT1P1*/*DKC1* exerts function on *FLT1* mRNA stability, we tested *FLT1* mRNA expression with the treatment of Actinomycin D. The results showed that *FLT1P1* knockdown decreased *FLT1* mRNA expression under Actinomycin D treatment (Figure 4a). We then silenced *DKC1* expression with the transfection of sh-*DKC1* in trophoblast cells for further analysis (Figure 4b). As shown, *FLT1* mRNA stability was significantly decreased by *DKC1* downregulation in HTR8 and BeWo cells treated with actinomycin D (Figure 4c). Furthermore, we found that the *FLT1* protein level in cells was also reduced by *DKC1* downregulation (Figure 4d). Overall, *FLT1P1* maintains *FLT1* mRNA stability via recruiting *DKC1*.

## *FLT1P1* regulates trophoblast cell proliferation and angiogenesis by mediating *FLT1*

**Figure 5.**
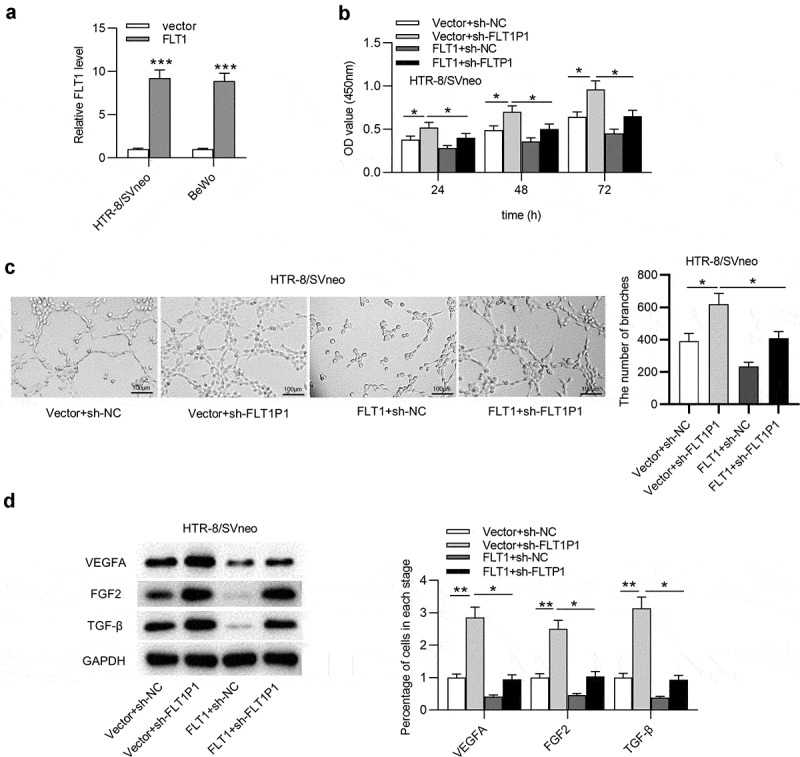
***FLT1P1* increases *FLT1* expression to regulate trophoblast cell proliferation and angiogenesis**. (a) The overexpression efficiency of *FLT1* was confirmed by RT-qPCR analysis. (b) The proliferative ability of trophoblast cells in each group was measured by CCK-8 assay. (c-d) Angiogenesis and angiogenesis-relevant protein levels were evaluated by tube formation assay and western blot analysis. *p < 0.05, **p < 0.01, ***p < 0.001

Finally, we verified whether *FLT1P1* regulates trophoblast cells by *FLT1*, and rescue assays were performed. The overexpression efficiency of *FLT1* was evaluated by RT-qPCR. The results showed that *FLT1* was effectively overexpressed with pcDNA3.1/*FLT1* transfection in HTR8 and BeWo cells (Figure 5a). As shown in CCK-8 assay, *FLT1* overexpression attenuated the promotive effect of *FLT1P1* knockdown on trophoblast cell proliferation (Figure 5b). We further discovered that angiogenesis restored by *FLT1P1* knockdown was inhibited by *FLT1* overexpression (Figure 5c). In addition, the VEGFA, FGF2, and TGF-β protein levels increased by *FLT1P1* knockdown was reduced after *FLT1* overexpression (Figure 5d). Collectively, *FLT1P1* upregulates *FLT1* expression to inhibit trophoblast cell proliferation and angiogenesis.

## Discussion

Pseudogenes, which are fairly common (~0.7% of DNA sequence) in the human genome, as well as lncRNAs are traditionally claimed to not yield functional mRNAs and not translated into proteins consequently, thus regarded as garbage fragments or dark matter in the genome [35]; however, evidence in recent years has indicated that pseudogene regulates various aspects of cell biology, and there is an increasing attention on its potential contribution to disease cause [36]. A previous study showed that the newly identified pseudogenes *BNIP3P1, HK2P1*, and *PGK1P1* that encode lncRNA are key PE-related genes [37]. Hexokinase 2 pseudogene 1 (*HK2P10*) expression is correlated with abnormal decidualization and might lead to the occurrence of PE [38]. However, the related mechanisms and the roles of abnormally expressed pseudogenes in PE have not been functionally characterized to date. Therefore, the identification of key pseudogenes associated with PE is critical to identifying novel therapeutic targets. A study pointed out that *FLT1P1* is expressed at a high level in preeclamptic placentas [26], implying that pseudogene *FLT1P1* might play an important role in the development of PE. The biological function of *FLT1P1* has not been investigated previously. In this study, we found that *FLT1P1* was upregulated in PTBs isolated from PE patients compared with healthy controls. The functional assays suggested that the downregulation of *FLT1P1* altered the proliferative capacity of HTR8 and BeWo cells. Defects in decidualization cause inadequate placentation and angiogenesis, which could give rise to PE [39]. VEGF is famous as an angiogenesis factor in many tissues and its decreased expression has been implicated in the pathophysiology of PE [40,41]. Here, it was demonstrated that *FLT1P1* knockdown promoted the angiogenesis in the trophoblast, demonstrating the key role of *FLT1P1* in PE.

Pseudogenes have a high sequence similarity to their parental protein-coding genes, which generates the potential for sequence-specific regulation [36]. The pseudogene *FLT1P1* shares molecular ancestry with the cognate gene *FLT1* (*VEGFR1*) in humans and high primates [25]. The VEGF family has been implicated as an important regulator of blood vessel formation in both health and disease states, including PE, tumor neovascularization, and diabetic retinopathy [42]. *FLT1* (*VEGFR1*) is a type V protein-tyrosine kinase receptor that is crucial for cell proliferation and differentiation and is expressed in vascular endothelial cells, placental trophoblast cells, and peripheral blood monocytes. A soluble form of *FLT1* is markedly increased during the last 2 months of gestation in those with PE compared with normotensive pregnant controls, which is involved in the endothelial dysfunction characterizing the pregnancy disorder of PE [43]. Here, our study demonstrated that *FLT1* was upregulated in PTBs isolated from PE patients. *FLT1* knockdown promoted the proliferation and angiogenesis in the trophoblast. One of the most commonly described biological feature of processed pseudogenes is the ability to influence the expression of their parental coding genes. The high sequence similarity between these RNA pairs sets up a certain level of competition for posttranscriptional regulators, including, among others, RBPs [44]. Thus, we further explored the regulatory relationship between *FLT1P1* and *FLT1*. Through bioinformatics analysis, *DKC1* was predicted to be an RBP interacting with both *FLT1P1* and *FLT1. DKC1* is a key RBP encoding a protein responsible for telomerase holoenzyme complex stability [45]. In our study, we confirmed that *DKC1* could bind to either *FLT1P1* or *FLT1*. Moreover, the knockdown of *FLT1P1* or *DKC1* effectively decreased the mRNA stability and protein level of *FLT1*. Therefore, we demonstrated that *FLT1P1* increases *FLT1* mRNA stability by recruiting *DKC1* in this study.

## Conclusion

In conclusion, we demonstrated that *FLT1P1* and *FLT1* play a vital role in PE by regulating trophoblast cell proliferation and angiogenesis. Moreover, *FLT1P1* increases *FLT1* mRNA stability via recruiting *DKC1*. These results show that dysregulated *FLT1P1* and *FLT1* may be related to the occurrence of PE, suggesting that *FLT1P1* and *FLT1* could act as useful biomarkers for the diagnosis of PE. The present study is not without limitations. First, clinical samples need to be collected from PE patients to further verify the clinical significance of our findings. Second, the related signaling pathways targeted by the *FLT1P1*/FLT1remain unclear and require further investigations.

## Supplementary Material

Supplemental Material
