# Supplementary figures and images for: Pseudogene fms-related tyrosine kinase 1 pseudogene 1 (FLT1P1) cooperates with RNA binding protein dyskeratosis congenita 1 (DKC1) to restrain trophoblast cell proliferation and angiogenesis by targeting fms-related tyrosine kinase 1 (FLT1) in preeclampsia
Source: Bioengineered. 2021 Oct 26;12(1):8885–97. doi: 10.1080/21655979.2021.1988366 (PMC8806956; doi:10.1080/21655979.2021.1988366)

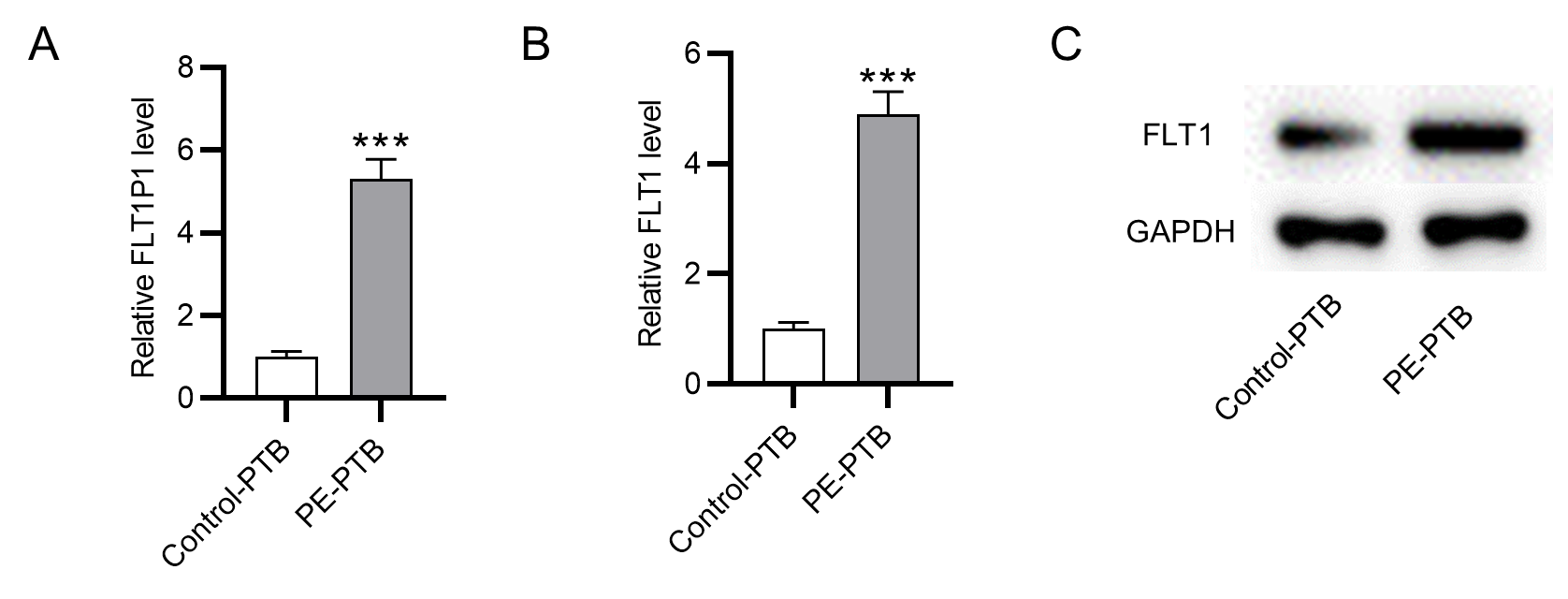

Supplement: Supplemental Material [file KBIE_A_1988366_SM4042.tif]
